# Reconstructive surgical treatment of peri-implantitis with use of a chitosan brush for decontamination- case series with 1-year follow-up

**DOI:** 10.1186/s40729-024-00574-7

**Published:** 2024-11-28

**Authors:** Gizem İnce Kuka, Hare Gürsoy

**Affiliations:** Department of Periodontology, Hamidiye Dental Faculty, University of Health Sciences, Istanbul, Turkey

**Keywords:** Peri-implantitis, Reconstructive surgery, Chitosan brush

## Abstract

**Introduction and aim:**

There is a need for studies assessing the efficacy of different therapeutic approaches in the reconstructive surgical treatment of peri-implantitis. The aim of this case series is to evaluate the clinical outcomes and radiographic bone fill of reconstructive surgical treatment using the oscilating chitosan brush for implant surface decontamination.

**Materials and methods:**

Nine patients with 11 Class I and III peri-implantitis defects were included. Following implant surface decontamination performed with a chitosan brush, guided bone regeneration (GBR) was performed by means of a bovine derived cancellous bone graft and collagen membrane. Clinical parameters such as full mouth plaque score (FMPS), probing depth (PD), bleeding on probing (BoP), recession, and radiographic bone level (RBL) were recorded at baseline and 1 year following treatment.

**Results:**

All evaluated parameters, including PD, BoP, recession, RBL, and full mouth plaque scores, revealed significant improvements at 1 year follow-up compared to baseline (*p < 0.05)*. The mean PD values reduced from 7.30 ± 1.29 to 3.78 ± 0.65 *(p = 0.000).* RBL was detected 5.50 ± 1.31 and 1.38 ± 0.74 at baseline and 1-year, respectively *(p = 0.010).*

**Conclusion:**

Reconstructive surgical treatment of Class I and III peri-implantitis defects with GBR may be an effective treatment protocol when an oscilitating chitosan brush is used for surface decontamination.

**Clinical trial number:**

Not applicable.

## Graphical Abstract

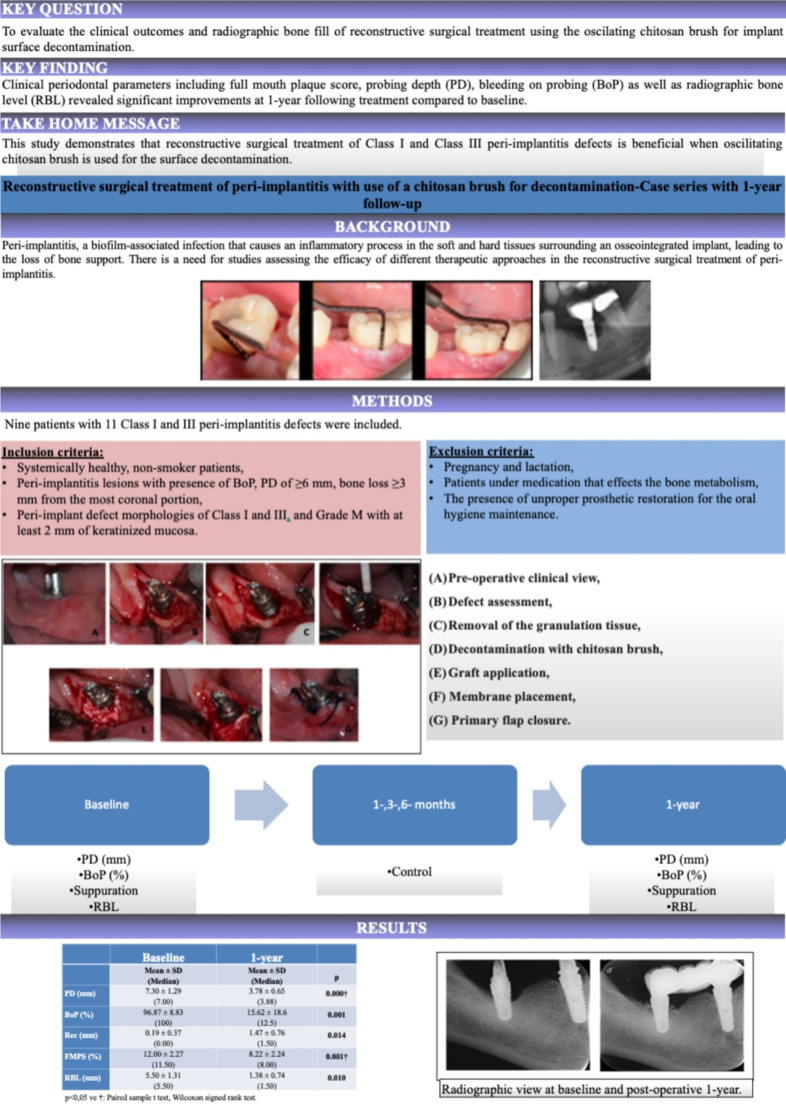

## Introduction

With the years’ progress in dental implantology, implant-supported restorations are regarded as the most sophisticated technique for partial and complete edentulous patients to restore lost teeth. As the understanding of dental implantology continues to improve, guidelines have been developed and will continue to be developed for long-term success. Despite the advances in implantology, there are some controversial topics and issues that are still subject to debate. One of the implant failures’ reasons is peri-implant disease. Especially peri-implantitis, a plaque biofilm-associated infection that causes an inflammatory process in the soft and hard tissues surrounding an osseointegrated implant, leading to the loss of bone support. Peri-implantitis has become a frequent complication that presents a challange to clinicians, with an increasing prevalence rate in clinical dental practice. Treatment of peri-implantitis aims to resolve inflammation and prevent further bone loss. Although non-surgical treatment alone may be effective for the suppression of inflammation in periodontitis, it has limited efficacy for moderate or advanced peri-implantitis lesions [1, 2]. Restricted access to the implant threads makes it challenging to properly clean the contaminated surface [3]. Many decontamination agents/methods have been reported as inadequate, and the presentation of an efficient decontamination procedure is still an active and open research area due to the paucity of information. Efficacy of mechanical implant surface decontamination methods such as ultrasonics, Er: YAG lasers, and air-abrasive devices with glycine powder has been investigated in previous clinical studies [4, 5]. None of these methods showed superiority in terms of PD and BoP reduction compared to conventional curettes [6]. It is of critical importance to develop devices or methods for proper implant surface decontamination. A non-toxic, biodegradable brush made of natural polysaccharide chitosan is reported to exhibit bacteriostatic and anti-inflammatory properties, and induce significant improvements in clinical parameters in the non-surgical treatment of peri-implantitis and peri-mucositis [7, 8]. However, its efficacy in surgical peri-implantitis treatment needs to be investigated.

In addition to decontamination, surgical treatment options, including resective or reconstructive techniques, may be required following non-surgical debridement. Surgical access following non-surgical therapy is mostly indicated to reduce the peri-implant PD’s and restriction of the inflammation. Different surgical approaches, including resective, reconstructive, and combined techniques, seem to improve treatment outcomes compared to non-surgical therapy alone [9–11]. In a recent systematic review, it was concluded that access flap surgery or reconstructive surgical techniques were both effective for the improvement of clinical parameters at 12-month follow-up; however, reconstructive techniques lead to improved radiographic outcomes. When peri-implantitis cases exhibit an intrabony defect of at least 3-mm component together with the presence of an adequate keratinized mucosa, reconstructive treatment modalities are suggested in the related guideline [11]. Reconstructive treatment modalities not only aim to augment the bone defect in the indicated cases but also limit peri-implant soft tissue recession.

Guided bone regeneration (GBR), a combination of grafts and barrier membranes, has been reported as a predictable treatment modality in surgical peri-implantitis treatment [9, 11–15]. Although autogenous grafts have been the gold standard, xenografts revealed better treatment outcomes in augmentation procedures in randomized clinical trials with long-term follow-up [15, 16]. When GBR is performed with a resorbable membrane, the risk of membrane exposure and infection is reduced, leading to more uneventful healing [14, 17]. Therefore, the aim of this case series is to evaluate the clinical outcomes and radiographic bone fill of reconstructive surgical treatment (a combination of a bone substitute; (Straumann^®^Cerabone^®^, Straumann AG, Basel, Switzerland), and a resorbable membrane; (Straumann^®^ Jason^®^membrane, Strauman AG, Basel, Switzerland), using the oscilating chitosan brush for implant surface decontamination.

## Material & methods

### Case selection

A prospective case series study was conducted according to the ethical principles of the Declaration of Helsinki and was approved by Hamidiye Scientific Research Ethics Committee (Decision Number 8/23). Inclusion criteria were: Having peri-implantitis lesions with presence of bleeding and/or suppuration on probing, probing depth (PD) of ≥ 6 mm, radiographic bone loss ≥ 3 mm apical from the most coronal portion of the intraosseous part of the implant, and having at least 2 mm of keratinized mucosa around the implant [18]. Patients with systemic diseases, pregnancy, and lactation, or under medication that effects the bone metabolism, without proper prosthetic restoration for oral hygiene maintenance and smokers were excluded. Before enrolment, written informed consent was obtained from all eligible patients.

Prior to the surgical stage, all patients received non-surgical periodontal treatment. Supra and subgingival debridement were performed with ultrasonics and hand instruments (Gracey and mini-five curettes). Oral hygiene education was given to each patient according to their needs. After 6 weeks, patients were recalled and enrolled in the study if their full-mouth plaque and bleeding scores were ≤ 20%. In total, 9 subjects aged between 30 and 65 years were included in the prospective case series. Peri-implant defect morphologies of Class I (infraosseous defect) and III (combined defet), and Grade M were treated [19]. All included patients completed the study period without dropouts.

### Surgical procedure

**Fig. 1:**
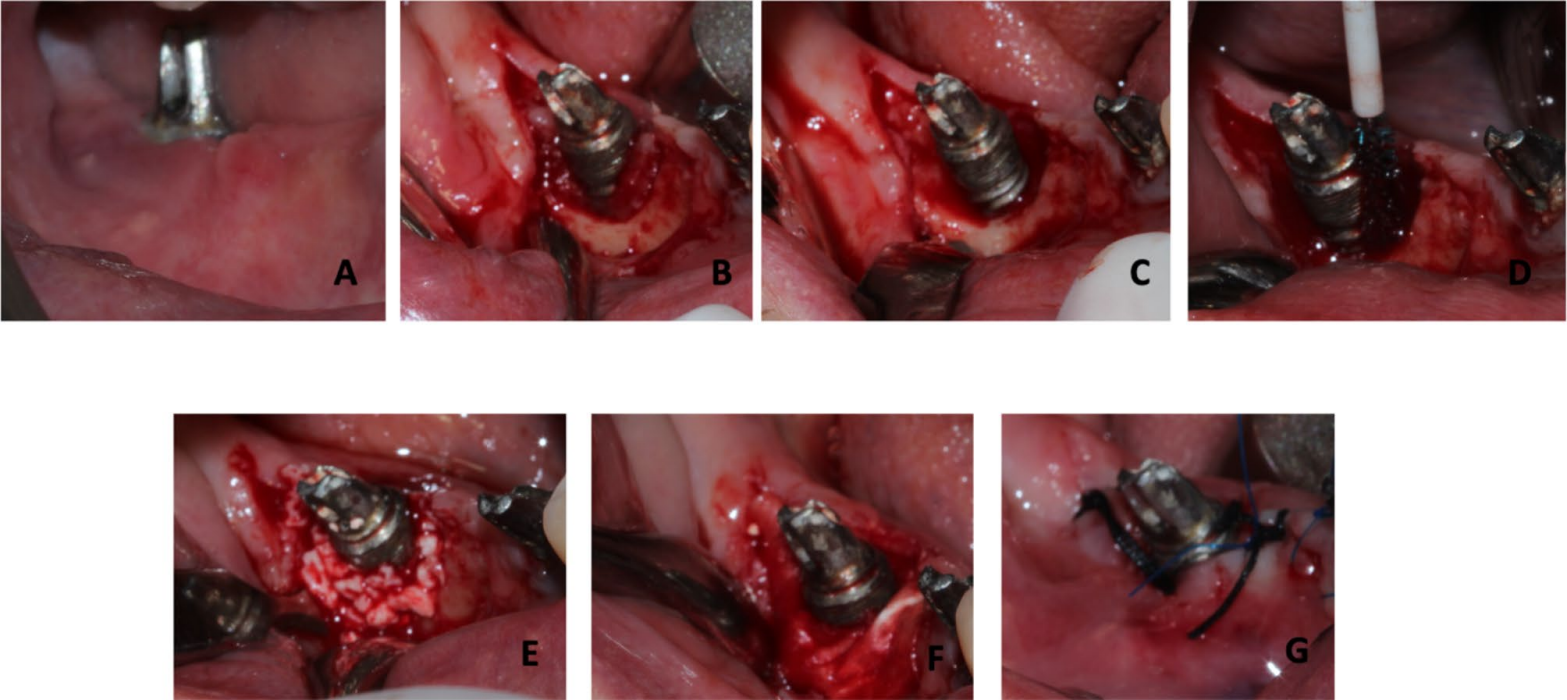
(**A**) Pre-operative clinical view, (**B**) Defect assessment, (**C**) Removal of the granulation tissue, (**D**) Decontamination of the implant surface with chitosan brush, (**E**) graft application, (**F**) membrane placement, (**G**) primary flap closure

**Fig. 2:**
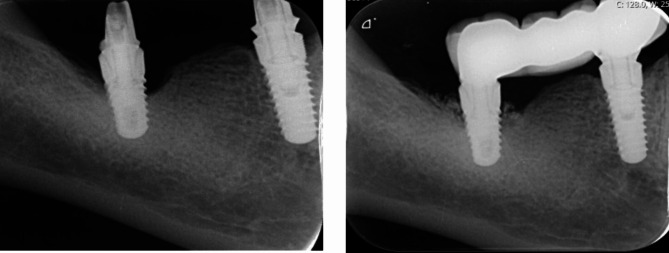
Radiographic view at baseline (left) and post-operative 12 months (right)

Following the application of local anesthesia, intrasulcular incisions were performed, and full- thickness flaps were elevated. Granulation tissues were removed with Ni-Ti curettes, and implant surface decontamination was performed with a chitosan brush (Labrida BioClean^™^, Straumann AG, Basel, Switzerland). After decontamination, the surgical sites were irrigated with a sterile saline solution to remove residues. Guided bone regeneration was performed with bovine derived cancellous bone graft with a particle size of 600–900 μm (Cerabone^®^, Straumann AG, Basel, Switzerland), and covered with Straumann^Ⓡ^ Jason^Ⓡ^ membrane, Strauman AG, Basel, Switzerland). Flaps were primarily sutured with 4/0 silk sutures (Propilen^®^, Dogsan, Trabzon, Turkey) (Figs. 1 and 2).

Patients received systemic amoxicillin + clavulanic acid (1 g twice a day for a week), non-steroid anti-inflammatory drugs (ibuprofen 600 mg, 1 tablet every 5–6 h for 5 days), and chlorhexidine mouth rinse initiated from the post operative 2nd day (twice daily for two weeks). After 2 weeks, the sutures were removed, and mechanical oral hygiene was resumed.

Patients were recalled at 1-, 3-, 6-months, and 1-year for supragingival professional prophylaxis.

The following parameters were evaluated at baseline and post-operative 1-year period:

- PD (mm) measured from six sites per implant with UNC probe (15 mm).
- Bleeding on Probing (BoP) (%): Evaluated at six sites per implant based on a dichotomous (0/1) scale, 10 s following PD measurements.
- Suppuration: Presence of suppuration/ pus formation was evaluated six sites per implant based on a dichotomous (0/1) scale, following PD measurements.
- Radiographic marginal bone level: Peri-implant marginal bone level (MBL) was measured from standardized periapical radiographs taken with the parallel technique (Kodak Dental System, Atlanta, USA). Measurements performed from mesial and distal parts of the implant platform to the crestal bone on each periapical radiograph were corrected according to the known height of each implant by using image analysis software (Image J, USA) [19, 20].

### Statistical analysis

Descriptive statistics (number, percentage, mean, standard deviation, median, minimum and maximum) of the data are given in Table 1. The assumption of normality was checked with the Shapiro Wilk test. Dependent Sample T test was perfomed to compare the means of two normally distributed dependent groups; In cases where the distribution was not normal, the Wilcoxon Sign Rank test was used. The McNemar test was used to examine the relationships between dependent categorical variables. Analyzes were carried out in IBM SPSS 25 program.

**Table 1:** Demographic data of the included-patients

AGE (Mean ± SD)	GENDER (F/M) (%)
51.8 ± 9.2	6/3 (66.7% ,33.3%)

## Results

A total of 11 implants from our 9 patients were treated in this prospective case series. Clinical and radigraphic data were recorded at baseline and 12-months post-operative. Mean age of the included subjects was 51.8 ± 9.2 years (66.7% females and 33.3% males) (Table 1).

The majority of the treated implants were located in the maxilla (64%). In particular, 18.1%, 45.4%, and 36.5% were in the anterior, premolar, and molar regions, respectively. Regarding morphology, 4 defects corresponded to Class I and the remaining 7 to Class III.

None of the treated cases revealed post operative complications such as infection and membrane exposure. No implant failures happened during the follow-up. Clinical and radiographic parameters significantly improved at the 12-month-period, shown in Table 2.

**Table 2:** Baseline and 1-year data of the evaluated parameters

	Baseline	1-year	
	Mean ± SD (Median)	Mean ± SD (Median)	*p*
PD (mm)	7.30 ± 1.29 (7.00)	3.78 ± 0.65 (3.88)	***0.000*** *†*
BoP (%)	96.87 ± 8.83 (100)	15.62 ± 18.6 (12.5)	***0.001***
Rec (mm)	0.19 ± 0.37 (0.00)	1.47 ± 0.76 (1.50)	***0.014***
FMPS (%)	12.00 ± 2.27 (11.50)	8.22 ± 2.24 (8.00)	***0.001*** *†*
RBL (mm)	5.50 ± 1.31 (5.50)	1.38 ± 0.74 (1.50)	***0.010***

*p* < 0,05 ve †: Paired sample t test

Wilcoxon signed rank test

## Discussion

The aim of a successful surgical peri-implantitis treatment is to resolve inflammation, decrease the bone defect, and limit peri-implant soft tissue recession [10–12]. The choice of surgical technique mostly depends on the defect morphology [10, 12, 20]. In the presence of horizontal bone loss, access flap or resective surgical techniques are indicated to sustain peri-implant tissue health and stable marginal bone levels. Reconstructive techniques for peri-implantitis treatment is indicated in 3-wall or circumferential defects with an intrabony component of 3 mm or more and an adequate keratinized mucosa around [11, 13]. Therefore, Class I and III peri-implantitis defects having at least 2 mm of keratinized tissue were included in the study.

During surgical treatment of peri-implantitis, implantoplasty has been suggested as an adjunctive measure to create a smooth surface to reduce biofilm accumulation [12]. Although this approach improved clinical and radiographic parameters significantly, it has also been associated with significant soft tissue recession, exposure of the implant surface, a limited esthetic outcome, and the implant corrosion [12, 21]. The anti-bacterial and anti-inflammatory effects of the chitosan brush have been shown in previous studies for the treatment of peri-mucositis and non-surgical peri-implantitis treatment [7, 8, 22]. However, no study has been conducted regarding the effectiveness of the chitosan brush in the surgical treatment of peri-implantitis for surface decontamination. Most of the previous studies that aimed to reconstruct the peri-implantitis defects used titanium curettes. The results of the present study are in line with those of previous studies reporting PD reductions around 3 mm and radiographic defect fill ranging between 1 and 5 mm within 1 year following the reconstructive peri-implantitis treatment [16, 17, 23, 24]. 

In a clinical study that used xenograft and collagen membrane in the surgical treatment of intra-bony peri-implant defects following the use of a rotary titanium brush, PD values reduced from 4.72 ± 1.02 to 3.18 ± 0.54 at 6-month follow-up [24]. In another study [25] that used a rotating titanium brush for decontamination, PD reduced by 2.92 ± 1.73 mm following deproteinized bovine bone mineral with 10% collagen application to defects at 1-year follow-up with improvements in BoP scores.

Previous studies have indicated that cone beam computed tomography (CBCT) has better sensitivity for fenestration and dehiscence defects but also has a tendency to underestimate or overestimate the size of defects compared to periapical radiographs. Moreover, periapical radiographs revealed better specificity in detecting peri-implant bone defects compared to computed tomography [19]. It was concluded that periapical radiographs should be used as a favorable method for peri-implant bone loss evaluation. [24, 25].

Successful surgical peri-implantitis treatment is defined as PD values around implant ≤ 5 mm, without suppuration and BoP and radiographic defect fill ≥ 1.0 mm [10, 20]. In the literature, several factors have been listed to influence treatment success negatively. One of those factors is inadequate post-operative plaque control [11, 12, 15, 26]. In order to eliminate the negative effects of the dental biofilm accumulation, the included patients were screened at 1-, 3-, 6-, and 12-month follow-ups for supragingival professional prophylaxis. FMPS remained below 10% throughout the post-operative period, which may explain the significant reductions of BoP and lack of suppuration in the present study results in addition to treatment success.

Given its limitations, including a limited number of treated peri-implantitis defects and the absence of a control group due to its case series design, one should interpret the present study findings with caution. Moreover, longer follow-up periods may be beneficial to determine the precise impact of this treatment approach and to evaluate the long-term stabilization of the graft material.

In conclusion, reconstructive treatment of peri-implantitis with the use of xenograft + resorbabale collagen membrane combination together with the chitosan brush for surface decontamination provided significant improvements in clinical and radiographic parameters and may be an effective treatment protocol. However, randomized, controlled, clinical trials with proper sample size calculations are required to compare the efficacy of different treatment modalities on different implant surface characteristics. Furthermore, the reconstructive potential of different peri-implant bone defect morphologies should be investigated.

## Data Availability

No datasets were generated or analysed during the current study.
